# Emergence of linezolid-resistant *Staphylococcus epidermidis* in the tertiary children’s hospital in Cracow, Poland

**DOI:** 10.1007/s10096-020-03893-w

**Published:** 2020-04-29

**Authors:** Maja Kosecka-Strojek, Ewa Sadowy, Iwona Gawryszewska, Joanna Klepacka, Tomasz Tomasik, Michal Michalik, Waleria Hryniewicz, Jacek Miedzobrodzki

**Affiliations:** 1 Department of Microbiology, Faculty of Biochemistry, Biophysics and Biotechnology, Jagiellonian University, Gronostajowa 7, 30-387 Krakow, Poland; 2 Department of Molecular Microbiology, National Medicines Institute, Warsaw, Poland; 3 Department of Clinical Microbiology, Children’s University Hospital, Jagiellonian University, Krakow, Poland; 4 MML Medical Centre, Warsaw, Poland; 5 Department of Epidemiology and Clinical Microbiology, National Medicines Institute, Warsaw, Poland

**Keywords:** Staphylococci, Antibiotic resistance, Infections, ICU

## Abstract

Coagulase-negative staphylococci, ubiquitous commensals of human skin, and mucous membranes represent important pathogens for immunocompromised patients and neonates. The increasing antibiotic resistance among *Staphylococcus epidermidis* is an emerging problem worldwide. In particular, the linezolid-resistant *S. epidermidis* (LRSE) strains are observed in Europe since 2014. The aim of our study was to genetically characterize 11 LRSE isolates, recovered mostly from blood in the University Children’s Hospital in Krakow, Poland, between 2015 and 2017. For identification of the isolates at the species level, we used 16S rRNA sequencing and RFLP of the *saoC* gene. Isolates were characterized phenotypically by determining their antimicrobial resistance patterns and using molecular methods such as PFGE, MLST, SCC*mec* typing, detection of the *ica* operon, and analysis of antimicrobial resistance determinants. All isolates were multidrug-resistant, including resistance to methicillin, and exhibited so-called PhLOPS_A_ phenotype. In PFGE, all isolates (excluding one from a catheter) represented identical patterns, were identified as ST2, and harbored the *ica* operon, responsible for biofilm formation. Linezolid resistance was associated with acquisition of A157R mutation in the ribosomal protein L3 and the presence of *cfr* gene. All isolates revealed new SCC*mec* cassette element composition. Recently, pediatric patients with serious staphylococcal infections are often treated with linezolid. The increasing linezolid resistance in bacterial strains becomes a real threat for patients, and monitoring such infections combined with surveillance and infection prevention programs is very important to decrease number of linezolid-resistant staphylococcal strains.

## Introduction

Coagulase-negative staphylococci (CoNS) ubiquitously colonize human skin and mucosal membranes, and due to this fact, they were for a long time considered harmless commensals [1]. Nowadays, however, they are increasingly important etiologic agents of hospital-acquired infections (HAIs), including central line-associated bloodstream infections (CLABSIs) and surgical-site infections (SSIs). Among human CoNS, *Staphylococcus epidermidis* represents the most frequently isolated species [2]. The increasing prevalence of antibiotic-resistant CoNS from nosocomial infections have been reported in Europe for some time [3–6], including especially worrisome methicillin-resistant *S. epidermidis* (MRSE) [7]. Since 2000, linezolid, a representative of the oxazolidynones has become an important addition in treatment for uncomplicated and complicated skin and skin structure infections and hospital- and community-acquired pneumonia caused by Gram-positive pathogens [8]. Shortly after the introduction into hospital practice, the first case of linezolid-resistant *Staphylococcus aureus* was reported in the USA in 2001 [9]. Linezolid-resistant *S. epidermidis* (LRSE) are increasingly observed in European countries, such as Portugal, Germany, Greece, Italy, Ireland, and France [10–17]. Linezolid resistance determinants may be acquired by staphylococci due to mutations selected during prolonged linezolid therapy and by horizontal gene transfer [18–21]. The G2576T mutation in the loop V of 23S rRNA is the principal determinant of the resistance; however, other mutations such as C2190T, T2502A, C2532T, and G2603T are observed as well [11, 22–24]. Mutations in the genes of ribosomal proteins L3 (*rplC* gene), L4 (*rplD* gene), and L22 (*rplV* gene) are also relatively frequently encountered among LRSE [8]. As reviewed by Mendes et al. [8], the alterations in L3 and L4 as a resistance mechanism appeared later in time and the complexity and number of such alterations in LRSE increased since 2014. The A157R modification in L3 was observed in the USA and Italy up to date [8]. Transferable genes conferring linezolid resistance in staphylococci include the *cfr* gene encoding ribosomal methyltransferase gene and the *optrA* and *poxtA* genes of ribosomal protection proteins [25–28]. Among these, only *cfr* was reported among LRSE so far [29]. Next emerging problem is that linezolid and methicillin resistance are often combined, so it is also important to characterize the SCC*mec* cassette elements in such isolates [16, 30–31].

Here, we present a report on a possible LRSE spread in the University Children’s Hospital (UCH) in Krakow, Poland, between 2015 and 2017. The aim of this study was to genetically characterize the LRSE strains, determine their clonal relationships, linezolid resistance mechanisms and refer the results to patients’ characteristics.

## Materials and methods

### Bacterial isolates

The study included 11 LRSE clinical isolates recovered between 2015 and 2017 from the UCH in Krakow from 10 pediatric patients aged from 23 days to 11 months. Nine isolates were recovered from blood (including two isolates from the same patient), one from a throat and one from a central venous catheter. All isolates were recovered after at least 48 h after admission to the unit. Linezolid resistance was detected in the hospital laboratory based on disc-diffusion method (linezolid (30 μg)). The preliminary identification of isolates was performed with BD Phoenix™ system (Becton Dickinson, Franklin Lakes, NJ, USA).

### Genomic DNA extraction

For genomic DNA extraction, isolates were grown for 18–20 h at 37 °C on blood agar plates. A full inoculation loop of 10 μl of bacterial colonies was homogenized with a TissueLyser II (Qiagen, Germantown, MD, USA). Total DNA was extracted by enzymatic lysis using the buffers and solutions provided with the DNeasy Blood and Tissue Kit (Qiagen, Germantown, MD, USA) according to manufacturer’s instructions.

### Species identification

All isolates were identified at the species level by sequencing of 16S rRNA as previously described [32] and PCR-RFLP of the *saoC* gene [33]. The 16S rRNA gene was amplified with slight modifications in PCR program: initial denaturation for 2 min at 94 °C, then followed by 25 cycles of denaturation at 94 °C for 30 s, annealing at 58 °C for 30 s, and extension at 72 °C for 60 s. The final extension was for 5 min at 72 °C. The PCR products were resolved by electrophoresis and purified using the DNA Clean & Concentrator™-5 purification kit (Zymo Research, Irvine, CA, USA). Two hundred nanograms of PCR product was used for sequencing with the primers used for PCR amplification. The *saoC* gene was amplified with the set of seven primers described previously [33]. PCR products were digested separately using each enzyme from the set of restriction enzymes (*Tai*I, *Tsp*509I, *Alu*I, and *Mse*I (Thermo Fisher Scientific, Waltham, MA, USA)). The obtained restriction patterns were compared to *saoC* gene fingerprint of the reference *S. epidermidis* strain.

### Susceptibility testing

Methicillin resistance was initially identified using cefoxitin disks (30 μg) (Oxoid Ltd., Cambridge, UK), and the phenotype was further confirmed by the detection of the *mecA* gene [34]. MIC values for linezolid, ceftarolin, vancomycin, teicoplanin, dalbavancin, daptomycin, and fosfomycin were determined using the Etest method (bioMérieux, Marcy l’Etoile, France); MIC of ciprofloxacin was determined by a broth microdilution method according to the European Committee on Antimicrobial Susceptibility Testing (EUCAST; www.eucast.org); for rifampin, tetracycline, minocycline, tigecycline, chloramphenicol, and gentamicin, the disk-diffusion method was used following the EUCAST recommendations. The results were interpreted using the EUCAST criteria. The *S. aureus* strain ATCC 29213 was used as a control. Inducible clindamycin resistance was tested by placing the erythromycin and clindamycin double-disk test according to the EUCAST recommendations.

### Molecular typing, analysis of antimicrobial resistance determinants, and detection of the *ica* operon

The clonality of isolates was studied using the pulsed-field electrophoresis (PFGE) of *Sma*I-digested bacterial DNA embedded in agarose plugs, as described by others [35] and the multilocus sequence typing (MLST) [36]. To assign alleles and sequence types (STs) for allelic profiles, the *S. epidermidis* MLST website (https://pubmlst.org/ sepidermidis/; 24th October 2019, date last accessed) was used [37]. The SCC*mec* cassettes were typed with two independent methods as described previously by Milheirico et al. [38] and Kondo et al. [34] with USA300 3956/13 strain as a positive control for IV SCC*mec* cassette.

Detection of the *cfr* and *optrA* genes was performed as previously, using strains from the laboratory collection as positive controls [39]. Presence of *aac(6′)-*Ie*-aph(2″)* and structure of Tn*4001* were studied as described [40] with the control strains characterized previously [41]. For the *poxtA*, *fexA*, *norA*, *fosB* and the *ica* operon detection by PCR and for *rplC*, *rplD*, *rplV* and the 23S rRNA gene sequencing primers were designed in the current study (Table 1).

**Table 1:** Primers used in the study

Gene	Sequence (5′-3′)
*poxtA*	TGCCCGTATTGGTTATCTCC
	TTCCTGCTCTGCATTGACTG
*fexA*	ATGACTCTGATGGGGCTGTC
	CCTGCTCCAAGGTACAAAGC
*norA*	CAAGGTTTTGCAGGTGGATTG
	TGCTTCTTTACGGCGTGACTT
*fosB*	AGGTGAGACCTCGGCCTAT
	CTTTCAACCAGATATACCAATCTTCA
*ica* operon	CTGGTAAAGTCCGTCAATGGAA
	TACCGTTGGATATTGCCTCTG
	ATTGACAGTCGCTACGAAAAGAAA
	ATCACTACCGGAAACAGCGAT
*rplC*	AGGAGGTGGACTTTCGATGAC
	TGCAATTTCCTCCTTTCGCTTC
*rplD*	TAAGAAGCGAAAGGAGGAAATTG
	ATTACGGGGCGCTTAAGAAC
*rplV*	TTTCAGCATACCATTTTGCTTCC
	TAAAGGACATGCAGCAGACG
23S rRNA	CGGCGGCCGTAACTATAACGCAGCACTTATCCCGTCCATAC

### Nucleotide sequence accession numbers

The 16S rRNA gene sequences were annotated using the NCBI BankIt tool and deposited in GenBank under accession numbers: MN850509–MN850519.

## Results

### Patient characteristics

**Fig. 1:**
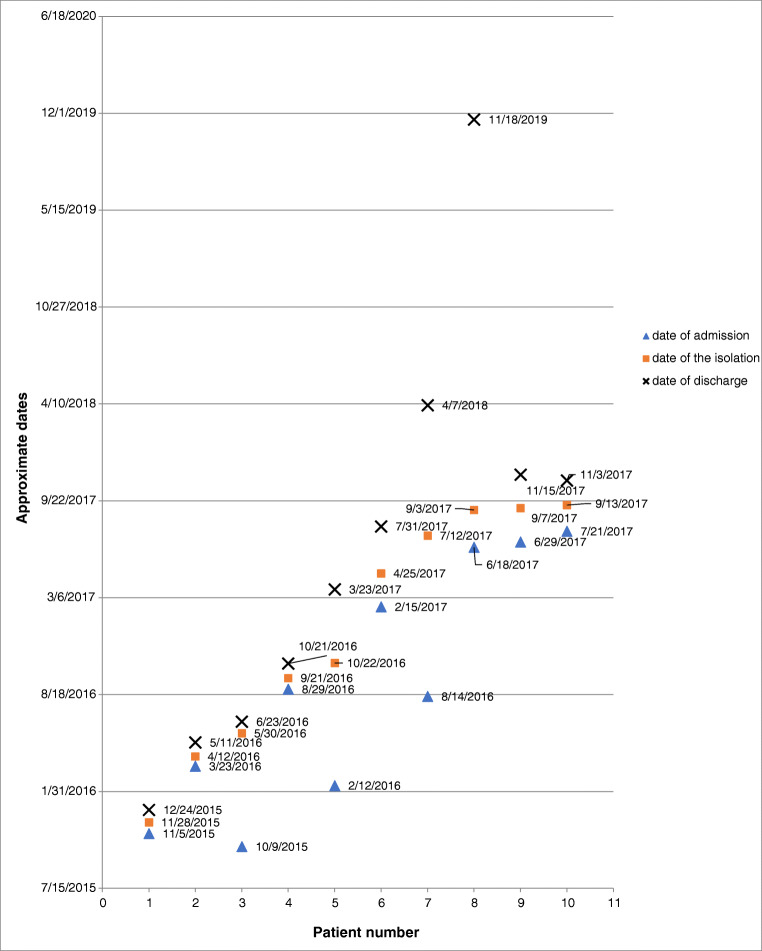
Dates of patient hospitalization and isolation of LRSE

We evaluated 10 patients (newborns and infants) age 23 days to 11 months at the time of the LRSE isolation; weight 915 to 6400 g on the day of admission to UCH. Four patients were small for gestational age (SGA), and no data was available for 2 patients. All patients were transferred from five different hospitals in Krakow or Lesser Poland. With an exception of one patient, all patients had at least one birth defect (Table 2). After transfer to the UCH, patients were admitted to two different Intensive Care Units: ICU 1 (*n* = 8) and ICU 2 (*n* = 2). All patients had indwelling central venous catheters. During the hospitalization, nine patients were transferred to another ward once or twice. The infection due to LRSE was diagnosed within 7 to 331 day after hospital admission. All LRSE isolates were recovered after at least 48 h after admission to the unit so the criterion of HAI is fulfilled. Patients were diagnosed with sepsis, fever, oliguria, and chronic respiratory failure. All patients underwent from one to six surgery operations. The values of infectious markers such as leukocytosis, the C-reactive protein (CRP), and procalcitonin (PCT) are presented in Table 2. At the time of LRSE isolation, six patients were treated with linezolid, but all patients were treated with linezolid at least once during hospitalization (Table 2). Figure 1 shows the time of patient hospitalization, LRSE detection, and date of patient discharge.

**Table 2:** Patients characteristics

Patient	Isolate no.	Date of isolate	Ward^a^	Age^a^ (days)	Weight^b^ (g)	Length of hospitalization^a^ (days)	Antimicrobial treatment^a^	No. of birth defects^b^	No. of surgery procedures^b^	Diagnosis associated with LRSE^a^ (sepsis etc)	Leukocytosis^a^ (leukocytes/mm^3^)	CRP^a^ (mg/l)	PCT^a^ (ng/ml)	Outcome
1	K/15/9696; K/15/9821	28.11.2015; 2.12.2015	ICU 1	23; 27	2900	23; 27	Linezolid (Zyvoxid); meropenem (Meronem); vancomycin	1	3	Fever; circulatory centralization; oliguria; edema	7180; 7250	57; 199.5	6.86; 1.98	Discharge
2	K/16/3213	12.04.2016	ICU 1	95	6400	7	Linezolid (Zyvoxid); meropenem (Meronem)	0	4	Fever	18,460	248	0.26	Discharge
3	K/16/4573	30.05.2016	ICU 2	236	3850	228	Ceftriaxone (Lendacin)	1	3	Fever	5100	8.2	n/d	Discharge
4	K/16/8122	21.09.2016	ICU 1	25	2740	15	Linezolid (Zyvoxid); meropenem (Meronem); vancomycin	3	1	The patient’s condition is stable, suspected asymptomatic endocarditis, no extra symptoms	4290	63	2.79	Discharge
5	K/16/9005	22.10.2016	ICU 1	268	915	15	Linezolid (Zyvoxid); meropenem (Meronem)	1	4	Severe chronic condition; respiratory failure; suppuration of the postoperative wound	17,340	55	0.11	Discharge
6	K/3119	25.04.2017	ICU 1	91	4820	68	Ceftolozane/tazobactam (Zerbaxa)	3	1	Very severe condition, but stable, no extra symptoms	8970	46.5	3.59	Discharge
7	K/17/5479	12.07.2017	ICU 1	337	2400	331	Vancomycin	3	6	Chronic severe condition, no extra symptoms	13,500	155.6	1.34	Death (not due to infection)
8	O/17/6502	03.09.2017	ICU 1	83	1880	76	Ceftazidime (Fortum); biseptol	1	3	Chronic respiratory failure, inflammation around the central catheter	7490	19	n/d	Discharge
9	O/17/6612	07.09.2017	ICU 2	70	3750	58	Linezolid (Zyvoxid); levofloxacin; colistin	2	3	Chronic circulatory failure; respiratory failure, no extra symptoms	10,130	65.8	0.28	Discharge
10	K/17/7152	13.09.2017	ICU 1	55	2300	54	Linezolid (Zyvoxid)	5	3	Sepsis; fever, gastrostomy suppuration	6670	245	0.24	Death (not due to infection)

*LRSE* linezolid-resistant *S. epidermidis*, *CRP* C-reactive protein, *PCT* procalcitonin

^a^At LRSE isolation

^b^Before LRSE infection

### Characteristics of isolates and species identification

A total of 11 isolates from blood (*n* = 9), throat (*n* = 1), and central venous catheter (*n* = 1) collected from pediatric ICUs patients were investigated. Two isolates (K/15/9696 and K/15/9821) were collected from the same patient, 23 and 27 days after hospitalization, respectively. The preliminary identification with BD Phoenix™ system identified seven isolates as *S. epidermidis* and three isolates as *Staphylococcus hominis*. The obtained 16S rRNA sequences, identical for all studied isolates, were analyzed using nucleotide BLAST (Basic Local Alignment Search Tool, http://www.ncbi.nlm.nih.gov/BLAST/) and aligned to the reference sequences deposited in the GenBank and leBIBI databases. Using the previously described criteria for *Staphylococcus* identification at the species level [42–43], the best and the second-best species alignments were analyzed, and all isolates were identified as *S. epidermidis*. The identification as *S. epidermidis* was additionally confirmed with the *saoC* gene restriction analysis.

### Molecular typing and *ica* detection

PFGE analysis included 10 isolates into the subtype 1A and the remaining isolate from a catheter into the related pulsotype 1B. All isolates represented ST2 and harbored the *ica* operon.

### Antimicrobial susceptibility and resistance determinants

Eleven investigated isolates fully shared their antimicrobial susceptibility profiles. All isolates remained susceptible to vancomycin, teicoplanin, daptomycin, ceftaroline, tetracycline, minocycline, tigecycline, rifampin, and erythromycin. All isolates showed high resistance to linezolid (MIC above 256 mg/L) together with resistance to chloramphenicol and clindamycin, consistent with the presence of *cfr*, rendering so-called PhLOPS_A_ phenotype [44]. No inducible clindamycin resistance was detected. The chloramphenicol-resistance gene, *fexA*, was also observed in the whole group. Additionally, the isolates demonstrated the A157R change in their deduced amino acid sequences of the L3 protein. No changes occurred in the analyzed part of 23S rRNA genes and in the L4 and L22 protein genes. The isolates also demonstrated resistance to methicillin, ciprofloxacin, fosfomycin, and gentamicin, and in agreement with these phenotypes, all isolates were positive for *mecA*, *norA*, *fosB*, and *aac(6′)-*Ie*-aph(2″)* located in the complete Tn*4001* transposon. Altogether, the isolates were resistant to six various classes of antimicrobials, i.e., they were multidrug resistant [45].

### SCC*mec* cassettes typing

In both SCC*mec* typing methods, all isolates revealed new composition of the SCC*mec* cassette. All LRSE strains harbored: *mecA*, *mecI*, SCCmecIII J1, and *dcs* genes which indicates the mixture of SCC*mec* type II and III cassette elements [38]. Despite the *dcs*, *mecI*, and *mecA* genes, the SCC*mec* II cassette includes *kdp* and *ccrB* genes which were not detected in LRSE isolates. Although, all 11 isolates had SCCmecIII J1 gene which is a component of SCC*mec* III but they did not harbor *RIF* gene. The Kondo typing method [34] showed that all 11 LRSE isolates possessed class A of *mecA* gene and two *ccr* complexes (*ccrAB3* and *ccrAB4*).

## Discussion

Linezolid is an effective treatment for multidrug-resistant Gram-positive bacteria and despite its broad use for almost 20 years, it still exhibits excellent activity against staphylococci. Linezolid resistance among *S. epidermidis* remains uncommon worldwide but the increasing resistance in European countries such as Greece, Spain, Portugal, Italy, France, and Ireland has been reported [10–17]. The LRSE outbreaks occur occasionally and are mainly associated with ICUs [15]. Here, we describe the first emergence of LRSE and MRSE in Poland in a pediatric ICU. The emergence of LRSE strains is associated with an increased prior linezolid usage. In our hospital, linezolid was introduced into practice in 2005. The first LRSE strain was isolated from cerebrospinal fluid at 12.01.2014 from the Oncology and Hematology Department. Since 2015, the increased number of LRSE was isolated from invasive infections with a highest number of isolates in 2017.

Pediatric patients are at particular risk of bacterial infections due to their immature immune system, and this risk is especially evident in premature newborns and babies undergoing medical procedures, such as surgery, presence of catheters, and prolonged/extensive antimicrobial treatment. Restriction of linezolid usage was associated with disappearance of the resistant strains from the affected ICU.

In Poland, the nosocomial *S. epidermidis* population is dominated by strains belonging to MLST clonal complex 2 (CC2) [http://eburst.mlst.net]. These clones are multiresistant, seem to persist in hospital environment, and evolve quickly due to mutations, recombination events, and frequent transmission of mobile genetic elements [46]. In the present study, all isolates belonged to ST2, a presumable ancestral type of CC2. In Germany, in 2015, Bender et al. described 12 (33%) LRSE belonging to ST2 [11], O’Connor et al. described 9 isolates (100%) as ST2 [13], and Barros et al. described one in a Portuguese hospital [10]. In 2018, Dortet et al. described an outbreak caused by ST2, ST5, and ST22 of LRSE in France [15]. In other European countries, five different STs were identified: (i) ST22 in Greece, Germany, and Spain [11–12, 16, 31]; (ii) ST23 in Italy and Germany [11, 14, 16]; (iii) ST83 in Italy [14]; and (iv and v) ST5 and ST168 in Germany [11, 16].

PFGE analyses are widely used for detection of the spread of a single clone at the local level [47], and we also applied this technique in our study. The restriction patterns of *Sma*I revealed two closely related PFGE types among isolates. Furthermore, all isolates shared the antimicrobial resistance phenotypes and determinants. Linezolid resistance was associated with acquisition of the A157R mutation in the ribosomal protein L3 and the presence of *cfr* gene. Such L3 alterations were described previously to impact linezolid susceptibility [48–49]. As shown in other studies, the *cfr* gene can co-occur with other linezolid resistance mechanisms [26, 50–51]. Importantly, the presence of a highly similar *cfr* plasmids in different genetic backgrounds was confirmed [17], and their acquisition via horizontal gene transfer in LRSE has been shown [11, 14]. The evidence for the presence of endemic LRSE clones that circulate in hospital settings was also reported [10, 12]. These strains differ from commensal *S. epidermidis* isolates and become more successful in the hospital environment [6]. All isolates carried also the *icaADBC* locus, which is responsible for the production of polysaccharide intercellular adhesin (PIA), playing an important role in formation of biofilm by the bacterium [52–53]. Since, the linezolid have been used to treat biofilm-associated *S. epidermidis* infections, the circulation of LRSE with a biofilm-associated operon constitutes a real threat for patients [54–55].

All studied isolates shared also the atypical composition of their SCC*mec* elements. Such situation is indeed observed in MRCoNS also by others [3, 56]. Chen et al. showed the multiple *ccr* complexes composition in CoNS strains. The authors showed the *ccrAB3* and *ccrAB4* genes in *S. hominis* and *S. capitis* [57]. Our study revealed not only the multiple *ccr* but also a combination of SCC*mec* cassette II and III elements in all our LRSE strains. The detection of new SCC*mec* cassettes composition of all LRSE strains revealed the acquisition of antibiotic resistance determinants within hospital environment and revealed that CoNS strains are a reservoir of antibiotic resistance genes which can be easily spread to *S. aureus* strains. To fully elucidate the structure of SCC*mec* element in studied isolates, the whole-genome sequencing is indispensable.

In conclusion, we have reported the first emergence of LRSE in Poland. Recently, linezolid is used more frequently, especially in pediatric patients for treatment of severe infections. As the ST2 LRSE is identified not only in Poland but in many European countries, it is very important to start or continue the surveillance, infection control, and antimicrobial guidelines against linezolid-resistant staphylococcal strains.
